# Workers of *Apis mellifera* Reared in Small-Cell Combs Show Higher Activity of the Proteolytic System in Hemolymph than Workers Reared in Standard-Cell Combs in Laboratory Cage Tests

**DOI:** 10.3390/ani13081368

**Published:** 2023-04-16

**Authors:** Piotr Dziechciarz, Aneta Strachecka, Grzegorz Borsuk, Krzysztof Olszewski

**Affiliations:** 1 Subdepartment of Apidology, Institute of Biological Basis of Animal Production, Faculty of Animal Sciences and Bioeconomy, University of Life Sciences in Lublin, 20-950 Lublin, Poland; 2 Department of Invertebrate Ecophysiology and Experimental Biology, University of Life Sciences in Lublin, 20-950 Lublin, Poland

**Keywords:** small-cell combs, *Apis mellifera*, hemolymph, proteolytic enzymes, protease inhibitors

## Abstract

**Simple Summary:**

Honeybees live in two different environments that are affected by multiple factors, e.g., pathogens or weather conditions. Thanks to the construction of the nests and behavioral cooperation honeybees became independent from the influence of external conditions. Moreover, a single bee has barriers that maintain homeostasis in its body and protect it from harmful factors, such as pesticides, air pollutants, or pathogens, through a system of proteases and their inhibitors. In feral bee colonies, bee comb cells vary in their width, while in Europe, colonies are kept only on standard-cell combs (5.50 mm cell width) or small-cell combs (4.90 mm cell width). In apiary conditions, the activities of proteases and their inhibitors in the 1-day-old workers were always higher in workers reared in standard-cell combs than in workers reared in small-cell combs, and opposite results were observed in groups of older workers (aged 7, 14, and 21 d). However, evaluating the influence of the nest factor is complex and difficult in field tests. Therefore, we assessed the proteolytic system activity in the hemolymph of colonies kept simultaneously on standard and small-cell combs in laboratory cage tests. The use of two types of combs in bee colony nests is an innovative approach to the use of small-cell combs in beekeeping. Irrespective of the age of the bees, the workers reared in the small-cell combs had higher protein concentrations. In turn, the activities of proteases and their inhibitors in the hemolymph of 1-day-old workers were higher in the group reared in the standard-cell combs. In older workers (aged 7, 14, and 21 days), higher protease and inhibitor activities were detected in the group reared in small-cell combs. The comparison of the results of apiary studies with those of laboratory cage tests shows that functions performed in the nest have a greater influence than age on the activity of proteases and their inhibitors in the hemolymph of worker bees.

**Abstract:**

The aim of the study was to compare the activities of proteases and their inhibitors in the hemolymph of honeybee workers reared in small-cell combs (SMC) and standard-cell combs (STC) in laboratory cage tests. The analyses conducted in laboratory conditions facilitated assessment of the impact of the comb cell width (small vs. standard) along with eliminating the influence of many environmental factors on the results. The width of the comb cells in which the workers were reared had a significant effect on the protein concentrations and proteolytic system activities in the hemolymph. Irrespective of the age of the workers, higher protein concentrations were found in the hemolymph of the SMC workers. In turn, the activities of proteases and their inhibitors in the hemolymph of 1-day-old bees were higher in the STC workers. In older bees, aged 7–21 days, activity was higher in the SMC workers. The role of the considerable cell width variability in natural combs that were built without the use of an artificially produced wax foundation is worth investigating. It is highly probable that the impact of the comb cell width on the features of workers reared in these combs modifies the age polyethism in the worker caste as well. The investigation results of one-season studies of honeybees could be seriously affected by random factors. To reduce the risk of these effects, it is advisable to continue experiments over a few consecutive years.

## 1. Introduction

Honeybees live in two environments which are characterized by substantially different parameters. One environment is the nest, where the conditions are regulated precisely by worker bees. Consequently, the colony/superorganism is independent of changing environmental conditions outside the nest [1]. The other living environment is the ecosystem outside the nest, which the bees cannot modify [2]. All resources required for the functioning of the colony are collected by workers, specifically foragers, from the environment surrounding the bee colony.

One of the adaptations of honeybees to living in two different environments is age polyethism in the worker caste, i.e., the performance of age-related functions in the colony [3]. A special stage in the specialization of workers is the transition from the function of nurse/nest workers to foragers [4]. Such a radical change in both the work environment and the type of work may result in considerable changes in the functioning of the nervous and immune systems [5,6,7]. Changes in the functioning of the immune system of workers are a result of the greater exposure of foragers to various environmental stressors present outside the nest.. Contaminants and pathogens encountered by worker bees in the environment surrounding the bee colony are transferred to their bodies and enter the nest with food and water, thus impacting the other bees and the brood in the colony [8,9,10,11]. Therefore, despite the precise regulation of the nest conditions, external environmental factors have a direct and indirect effect on colony functioning [12,13]. The quality and availability of resources as well as the amount and type of pathogens and contaminants transported to the nest determine the biological quality of the bees [14,15], their lifespan, and their work performance. Such a complicated network of dependencies impedes assessment of, e.g., the genetic determinants of bee traits and the impact of factors present in the external and internal nest environment on bees [16,17]. Hence, such assessment in apiary/colony conditions is difficult to conduct and elucidate. [16,18,19]. To reduce the randomness of environmental factors and determine the impact of a specific factor/substance, laboratory cage tests are used in research on honeybees [20,21,22,23,24].

The wax comb built by bee workers, which consists of numerous cells, is the largest structural element of the colony nest. However, the structure of bee colony nests that are currently kept by humans differs significantly from original nests built by wild-living colonies. At present, combs in developed countries are built on thin beeswax sheets bearing imprinted cell outlines, i.e., wax foundations produced artificially as a template for honeybees to build comb cells.

It is believed that the main effect of introducing the wax foundation was the increase in the width/size of worker comb cells [25]. Currently, the standard width of worker cells in Europe varies within a small range, usually between 5.40 and 5.50 mm [25,26]. In contrast, the width of worker comb cells in natural nests of European dark bees *Apis mellifera mellifera* was found to range from 4.90 to 5.10 mm [27]. Comb cells with a width of 4.90 mm are referred to as “small cells” [25]. However, the standardization of the cell width/size itself in wax foundations was as important as enlarging the comb cell’s width as it substantially limited the variability in the comb cell width within the colony. Maggi et al. [28] measured the width of cells in colony nests with natural combs built without the use of the wax foundation and reported a considerable variability in this parameter. Worker brood and drone brood were reared in cells with widths of 4.17–6.75 mm and 5.04–8.05 mm, respectively.

Given the large variability of the comb cell width in a bee colony indicated by Maggi et al. [28] and the thesis proposed by Tautz [1], who described the honeycomb as the most important organ of the colony superorganism based on the diversity of its functions, we were the first to conduct pioneer studies focused on elucidation of the role of such a large variability in the comb cell width in the same bee colony. We introduced two types of combs in colony nests and kept the colonies on both small and standard cells at the same time. This meant that the variability of the comb cell width in the nests was similar to that found in nests built without the use of wax foundations [28]. Our previous studies showed that female workers reared in the so-called small cells (width: approx. 4.90 mm) differed from those reared in standard cells (width: approx. 5.50 mm) in their morphological [29] and physiological [30] traits. The width of comb cells may also modify the intensity of hygienic behavior in colonies, i.e., removal of dead brood, which affects the behavior of workers [31,32]. In apiary conditions, comparing the activity of the proteolytic system in the hemolymph of workers reared in small- and standard-cell combs allowed us to hypothesize that workers reared in small cells are predisposed to serve as foragers, and those reared in standard cells tend to work within the nest e.g. as serving the function of nurses [30]. In our previous work, we found that, in apiary conditions, the activity of the proteolytic system in the hemolymph of older workers (aged 7 d, 14 d, and 21 d), as expressed by the activities of proteases and their inhibitors, were always significantly higher in workers reared in small cells than in those reared in standard cells [30]. Opposite results were shown for the protein concentrations, where activities were always significantly lower in workers reared in small cells than in those reared in standard cells at the age of 7–21 days.

The aim of this study was to assess changes in the activity of the proteolytic system in the hemolymph of worker bees that were reared in cells with different width values (small vs. standard) in laboratory cage tests. The investigations conducted under laboratory conditions allowed the effect of the comb cell width to be independently assessed by eliminating the influence of numerous environmental factors on the results, which is not possible in apiary studies [30]. The assessment of proteolytic system parameters in laboratory cage tests can shed new light on the biological bases of beekeeping management technology involving the usage of small cell combs.

## 2. Materials and Methods

The experiment was conducted at the University of Life Sciences in Lublin (Lublin, Poland). The workers used in the laboratory cage tests were reared in the University apiary (51.224039 N–22.634649 E). A long-term study was conducted over three consecutive years (2020, 2021, and 2022) to omit random effects of environmental factors.

### 2.1. Acquisition of Bees

The group of foster colonies (*n* = 5) kept in Dadant Blatt hives were analyzed each year. The colonies had similar strength and structure, with nests containing eight brood combs, one drone comb, and one storage comb, all of which were fully populated by workers and naturally inseminated sister queens of the same age [30]. The nest of each foster colony contained two types of combs: standard-cell (cell width: approx. 5.50 mm) and small-cell (cell width: approx. 4.90 mm) combs [30]. The small- and standard-cell combs arrangement of the brood chamber was consistent with that reported by [32]. The workers in each foster colony were reared on a small-cell comb (SMC) and a standard-cell comb (STC) [30].

### 2.2. Laboratory Cage Tests

Workers were collected from all of the foster colonies and from each type of comb (small/standard cells) and were transferred to three cages. Two groups of cages were created in this way; one group was composed of SMC workers (reared in small-cell combs) and the other group comprised STC workers (reared in standard-cell combs). Each group consisted of 15 cages (5 colonies with 1 SMC comb and 1 STC comb in each × 3 cages of each type of comb = 15 cages with SMC workers and 15 cages with STC workers). The cages were kept in controlled conditions of 25 °C with ad libitum access to sugar syrup, which was composed of water and sucrose in a 1:1 ratio. Each day, the syrup was replaced with a fresh supply and dead bees were removed.

### 2.3. Hemolymph Collection and Analysis

Hemolymph was collected on days 7, 14, and 21 from the SMC and STC workers. No hemolymph was sampled from 21-day-old workers in 2020 as the bees in all cages had died before that time. The hemolymph was collected at the site of antenna detachment [33]. As in Borsuk et al. [33], tweezers were used to detatch antennas, and an automatic pipette with sterile tips was used to collect the hemolymph. One hemolymph sample was collected from five randomly selected worker bees from each cage and each group (SMC/STC). Hemolymph was collected from 1-day-old workers from each foster colony and each type of comb (small cells/standard cells) in the same way.

Each hemolymph sample was transferred into a separate Eppendorf tube (0.5 mL) that was filled with 150 µL of 0.6% NaCl, and then placed in a cooling block to prevent melanization [24,30,34]. Immediately after hemolymph collection, the tubes were frozen and stored at −80 °C. The number of hemolymph samples collected in each year is shown in Table 1. To eliminate the impact of potential *Nosema* spp./*Variomorpha* infection of the workers on the results, each group of sample worker bees was checked for the presence of *Nosema* spp. spores [35].

Total protein concentrations were assayed with the Lowry et al. [36] method as modified by Schacterle and Pollack [37]. The activities of acidic (pH 2.4), neutral (pH 7.0), and alkaline (pH 11.2) proteases in the hemolymph of the bees were analyzed with the Anson [38] method as modified by Strachecka et al. [39]. The activities of acidic (pH 2.4), neutral (pH 7.0), and alkaline (pH 11.2) protease inhibitors in the hemolymph were determined using the Lee and Lin method [40].

### 2.4. Statistical Analysis

The statistical analysis of the results was carried out using Statistica software formulas, version 13.3 (2017) for Windows, StatSoft Inc., Tulsa, OK, USA.

The effect of the year (2020, 2021, and 2022) and age (1 d, 7 d, 14 d, and 21 d) in each study year on the protein concentration and activity of the analyzed types of proteases and their inhibitors were assessed separately for the SMC and STC workers. ANOVA was used for normally distributed data and the Kruskal–Wallis test was employed for non-normally distributed data. The distribution of these data was analyzed using the Kolmogorov–Smirnoff test.

The protein concentrations and the activities of each type of protease (acidic, neutral, and alkaline) and its inhibitors in 1-day-old bees were compared between the SMC and STC workers using the *t*-Student test for data with normal distribution, and the pairwise Wilcoxon test was used for data with non-normal distribution. The distribution of these data was analyzed with the Shapiro–Wilk test.

The protein concentrations and the activities of each type of protease and its inhibitors within the age groups (7 d, 14 d, and 21 d) were compared between the SMC and SMC groups using the *t*-Student test for independent samples and the Mann–Whitney U test for data with non-normal distribution. The distribution of these data was analyzed using the Shapiro–Wilk test.

The effect of the year (2020, 2021, 2022) on the width of comb cells in the foster colonies was analyzed separately for small-cell combs (*n* = 300) and standard-cell combs (*n* = 300) using the Kruskal–Wallis test. The distribution of these data was analyzed with the Kolmogorov–Smirnoff test. The width of the small-cell combs in the foster colonies (*n* = 300) was compared with the width of the standard-cell combs (*n* = 300), collectively, for the three years using the Mann–Whitney U test, as the effect of the year was not significant for either the small- or standard-cell combs. The distribution of these data was analyzed using the Kolmogorov–Smirnoff test.

## 3. Results

### 3.1. Comb Cell Width

The were no significant differences in width of cells in the small-cell and standard-cell combs of the foster colonies between the years (respectively: H = 0.28, df = 2, *p* = 0.87, *n* = 300; H = 2.39, df = 2, *p* = 0.30, *n* = 300; Kruskal–Wallis test).

The width of the small-cells was significantly smaller (*p* ≤ 0.01; *n* = 300; Mann–Whitney U test) than that of the standard-cells. The mean values of the width of small-cells in the combs of the foster colonies was 4.97 mm (SD = 0.04), whereas the width of standard-cells in the combs of the foster colonies was 5.56 mm (SD = 0.05).

### 3.2. Protein Concentrations, Protease, and Protease Inhibitor Activities

No spores of *Nosema* spp./*Variomorpha* were found in any of the worker bees in the laboratory tests. Therefore, it was assumed that the *Nosema* spp./*Variomorpha* infestation did not influence the results of the experiments.

In both the SMC and STC worker groups, the year exerted a significant effect on the protein concentrations and the activities of all types of protease inhibitors (acidic, neutral, and alkaline) (Table 2). The effect of the year on the activities of all types of proteases (acidic, neutral, and alkaline) in the SMC workers were not significant. In turn, a significant effect on the activities of alkaline proteases exclusively were found in the group of the STC workers (Table 2). In all study years, the age (1 d, 7 d, 14 d, and 21 d) had a significant effect on all hemolymph parameters in the SMC and STC workers (Table 2).

**Figure 1:**
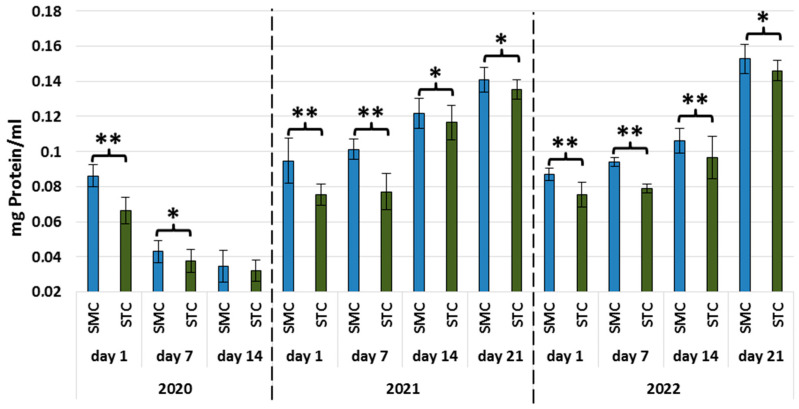
Protein concentrations in the hemolymph of workers in three consecutive years. SMC—workers reared in small-cell combs; STC—workers reared in standard-cell combs; *—differences between SMC and STM within the age group are significant (*p* ≤ 0.05); **—differences between SMC and STM within the age group are significant (*p* ≤ 0.01); vertical bars indicate standard deviation.

With the exception of the 14-day-old workers in 2020, the protein concentrations were significantly higher (*p* ≤ 0.01) in the SMC vs. STC workers in all age groups (1 d, 7 d, 14 d, 21 d) in all years (2020, 2021, and 2022) (Figure 1, Table 3).

**Figure 2:**
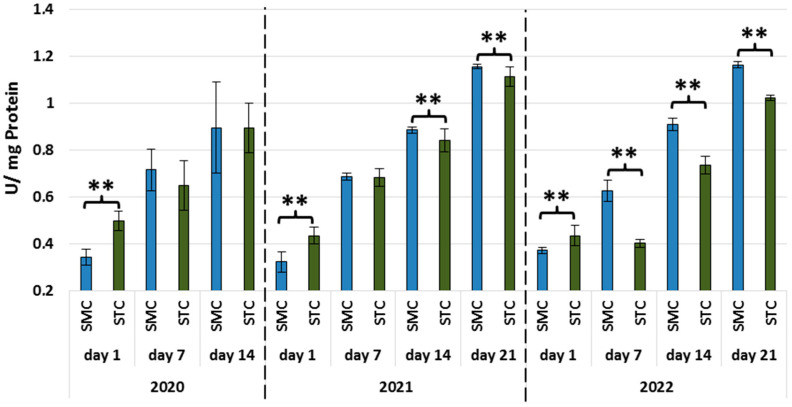
Acidic protease activities in the hemolymph of workers in three consecutive years. SMC—workers reared in small-cell combs; STC—workers reared in standard-cell combs; **—differences between SMC and STM within the age group are significant (*p* ≤ 0.01); vertical bars indicate standard deviation.

**Figure 3:**
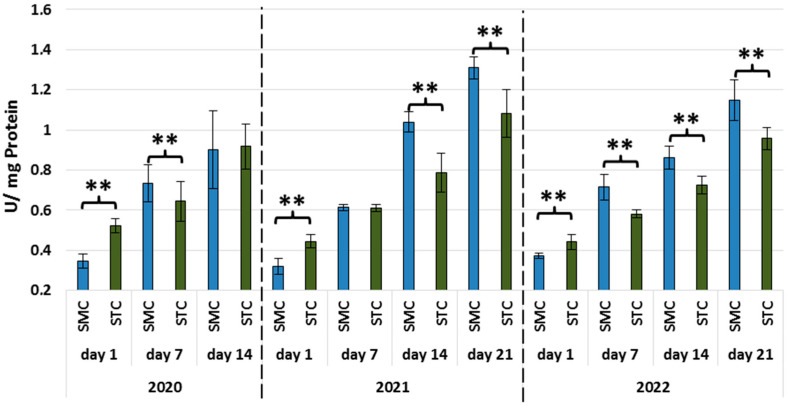
Neutral protease activities in the hemolymph of workers in three consecutive years. SMC—workers reared in small-cell combs; STC—workers reared in standard-cell combs; **—differences between SMC and STM within the age group are significant (*p* ≤ 0.01); vertical bars indicate standard deviation.

**Figure 4:**
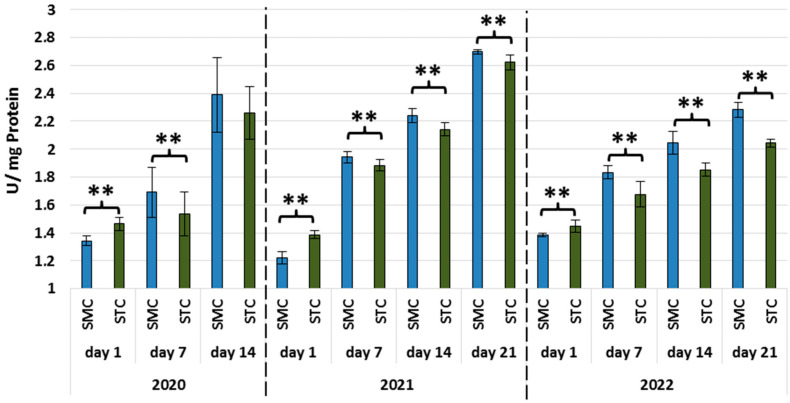
Alkaline protease activities in the hemolymph of workers in three consecutive years. SMC—workers reared in small-cell combs; STC—workers reared in standard-cell combs; **—differences between SMC and STM within the age group are significant (*p* ≤ 0.01); vertical bars indicate standard deviation.

**Figure 5:**
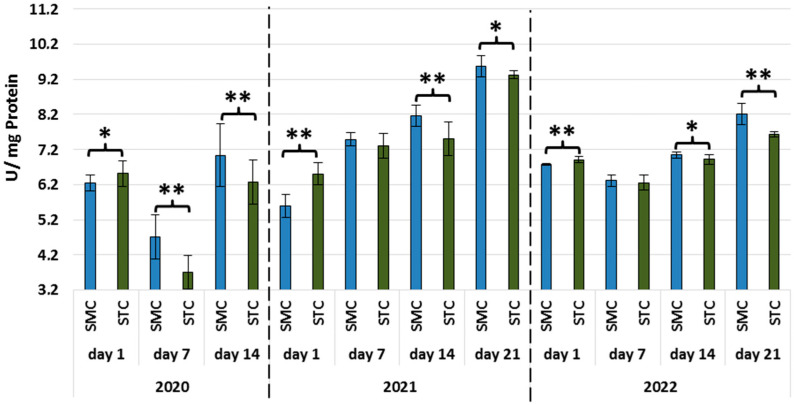
Acidic protease inhibitor activities in the hemolymph of workers in three consecutive years. SMC—workers reared in small-cell combs; STC—workers reared in standard-cell combs; *—differences between SMC and STM within the age group are significant (*p* ≤ 0.05); **—differences between SMC and STM within the age group are significant (*p* ≤ 0.01); vertical bars indicate standard deviation.

**Figure 6:**
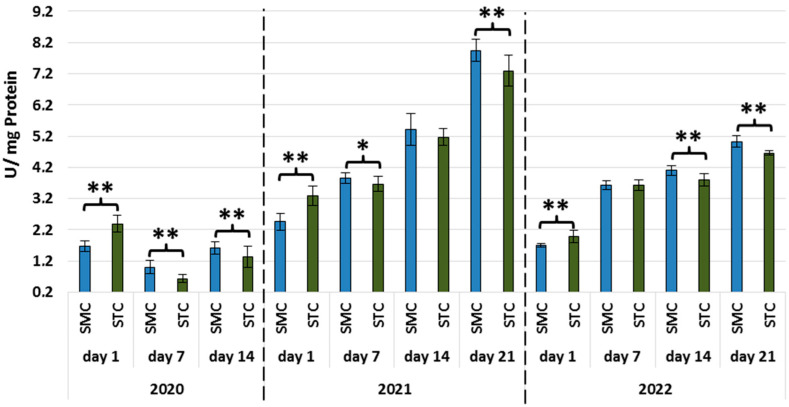
Neutral protease inhibitor activities in the hemolymph of workers in three consecutive years. SMC—workers reared in small-cell combs; STC—workers reared in standard-cell combs; *—differences between SMC and STM within the age group are significant (*p* ≤ 0.05); **—differences between SMC and STM within the age group are significant (*p* ≤ 0.01); vertical bars indicate standard deviation.

**Figure 7:**
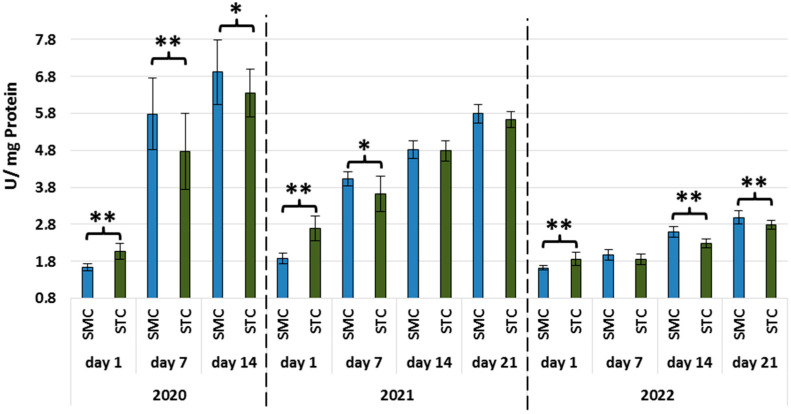
Alkaline protease inhibitor activities in the hemolymph of workers in three consecutive years. SMC—workers reared in small-cell combs; STC—workers reared in standard-cell combs; *—differences between SMC and STM within the age group are significant (*p* ≤ 0.05); **—differences between SMC and STM within the age group are significant (*p* ≤ 0.01); vertical bars indicate standard deviation.

In all years, the activities of all types of proteases and protease inhibitors were statistically significantly higher in the 1-day-old STC vs. SMC workers (Figure 2, Figure 3, Figure 4, Figure 5, Figure 6 and Figure 7, Table 3). In contrast, the older workers (7 d, 14 d, 21 d) exhibited an opposite trend, i.e., the activities were higher (mostly with statistical significance) in the SMC workers. The activities of proteases and their inhibitors in the SMC and STC workers were similar only in a few cases (Figure 2, Figure 3, Figure 4, Figure 5, Figure 6 and Figure 7, Table 3).

## 4. Discussion

As in our previous apiary experiments, the comb cell width exerted a significant effect on the protein concentrations and the activities of proteases and their inhibitors in the hemolymph of worker bees [30].

The same trend in the protein concentrations was recorded on the workers’ first day of life, and the value of this parameter was higher in the SMC workers in the apiary [30] and laboratory conditions (Figure 1, Table 3). In turn, the protein concentrations in the older workers (7 d, 14 d, 21 d) analyzed in the laboratory cage tests were significantly higher in the SMC vs. STC group. An opposite trend was recorded in the apiary conditions [30], where the protein concentrations in the SMC group were significantly lower than in the STC workers. Given these statistically significant differences in the protein concentrations between the SMC and STC workers, the structure of the fat body should be compared, with consideration of the segmental character that was recently discovered [41].

The comparison of the results of apiary and laboratory tests conducted in our present and previous studies indicates a significant effect on the traits of worker bees when they are reared in small-celled combs. In the apiary conditions, the SMC workers had a lower protein concentrations in the hemolymph but higher activities of proteases and their inhibitors than the STC workers [30]. The SMC workers exhibited higher catalase and superoxide dismutase activities as well as total antioxidant capacity in the hemolymph than the STC workers in both laboratory and apiary conditions [42]. Moreover, the SMC workers lived longer than the STC bees in the laboratory conditions [21].

The impact of rearing workers in small-cell combs or the use of such combs in the apiary is not limited only to the values of worker traits; it also modifies the traits of the bee colony. Colonies kept on small-cell combs exhibit more intensive hygienic behavior in terms of dead brood removal than those kept on standard-cell combs [31]. Dead brood is removed more effectively from small- than standard-cell combs [32]. Additionally, colonies with both small- and standard-cell combs in the nest (simultaneous use of two types of combs) have a higher performance value, as they develop faster in spring and produce significantly greater amounts of honey than colonies kept on only one type of comb, i.e., either with only small cells or only standard cells [43].

Table 4 compares the trends in the changes to the hemolymph parameters that occur with age in the SMC and STC workers as were assessed in the present laboratory cage tests and the previous study conducted in apiary conditions [30]. The protein concentrations increased with age in both the SMC and STC workers in the laboratory cage tests. In the apiary conditions, the value of this parameter decreased in the SMC workers and increased in the STC group.

The age-related decrease in the protein concentrations in the hemolymph of the SMC workers in the apiary conditions was associated with their transition from nurse/nest workers to foragers [30]. We assumed that SMC workers started working as foragers earlier and more frequently, while STC workers more often work in the nest, e.g., as nurse workers, as the protein concentrations in the hemolymph of STC workers increased with age [30]. The basis for this inference is the finding of lower protein concentrations in the hemolymph of foragers vs. nurse workers [44,45,46,47]. The bodies of nurse bees also contain greater amounts of protein [48]. The studies cited previously indicate that the protein concentration in the hemolymph is one of the physiological markers that distinguishes foragers from nurses.

The hypothesis that SMC workers serve as foragers was also confirmed by the results of the laboratory cage tests, where the protein concentrations in their hemolymph increased with age, as in the case of the STC workers. This was probably related to the fact that workers kept in cages could not undertake any specialized work and did not utilize the protein stored in their hemolymph and fat body. In turn, in apiary conditions, the highest protein concentrations were detected in larvae and pupae, lower levels were found in nurse workers, and the lowest amounts were found in foragers [44,45]. The differences in the age-dependent changes in the protein concentration in laboratory vs. apiary conditions are probably associated with the absence of the modifying impact of nest environment factors and external environment in laboratory cage tests.

In the SMC workers examined in the laboratory and apiary conditions, the activities of proteases in the hemolymph increased with age (Table 4). The values of these parameters in the STC group increased in the laboratory cage tests but persisted at a similar levels in the apiary conditions.

The higher activities of proteases and their inhibitors in the hemolymph of the SMC workers in the laboratory cage tests may indicate that they are more predisposed to work as foragers than the STC workers. The higher activities of proteases and their inhibitors in the hemolymph of the SMC workers may result from their enhanced exposure to numerous immunosuppressive factors encountered outside the nest. The activity of the proteolytic system, which is one of the measures of humoral immunity in the hemolymph, is influenced by the following factors: pesticides [49,50,51], biostimulants [52,53], and caste status [54].

The predisposition of SMC workers to serve as foragers has been confirmed in morphological studies. As reported by Vance et al. [55], foragers have lower body weight than nurses. This was confirmed in a study conducted by [29], where the body weight of SMC workers was 7.1% lower than that of STC workers, while the wings of the former bees were only 3.4% shorter and 2.8% narrower. In turn, the larger body size may predispose STC workers to serve as nurses, as such bees are regarded to be better nurses than smaller ones [56]. Additionally, STC workers have a significantly larger head (width and height) than SMC workers [29], which may be associated with the size and efficiency of their hypopharyngeal glands. In these glands, nurses produce royal jelly, which is food for larvae. Further investigations of STC and SMC workers hypopharyngeal glands must be conducted. Our ongoing research focuses on measuring STC and SMC workers’ hypopharyngeal glands and comparing the number of marked foragers and the location of marked workers in the nest (open brood, capped brood, and storage combs) to clarify the predispositions of workers reared in different type of combs to different tasks in the colony.

Based on the information presented in this discussion, it can be assumed that the width of the comb cells has an impact on the division of labor in the worker caste. Given this assumption, it can be hypothesized that, along with age polyethism, the significant variability in the width of comb cells in nests built without a wax foundation [28] introduces components of morphological polyethism into the bee colony. Morphological polyethism is typical of some ant species [57]. The combination of age polyethism in a bee colony with elements of morphological polyethism may indicate a compromise between strict specialization and behavioral flexibility. However, further investigations are required to corroborate this hypothesis.

The results of the protein concentrations and activities of protease inhibitors in 2020 differed significantly from those obtained in the other two years. Since the cages in the laboratory tests were kept in the same conditions, i.e., in a room with a constant temperature, throughout the three study years, we assume that this may have been caused by an unidentified factor that occurred during the rearing of worker bees in foster colonies. Thus, one-season studies of honeybees are burdened with the risk of random factors affecting the results. Therefore, to reduce this risk, it is advisable to continue such investigations over a few consecutive years.

## 5. Conclusions

The width of comb cells where worker bees were reared had a significant effect on the protein concentrations and the activity of the proteolytic system in the hemolymph of workers kept in the laboratory conditions. Irrespective of the age of the bees, the workers reared in the small-cell combs had higher protein concentrations. In turn, the activities of proteases and their inhibitors in the hemolymph of 1-day old workers were higher in the group reared in the standard-cell combs. In older workers (aged 7, 14, and 21 days), higher activities were detected in the group reared in small-cell combs.

One-season investigations of honeybees pose a risk of a significant effect exerted by random factors on the results. Therefore, continuation of research over a few consecutive years is recommended to reduce this risk.

The comparison of the results of apiary studies with the results of laboratory cage tests shows that functions performed in the nest have a greater influence than the age on the activity of proteases and their inhibitors in hemolymph of worker bees. This was especially noticeable in the case of workers reared in the standard-cell combs.

It is worth explaining the mechanisms and factors causing the construction of cells of different sizes in the nest of the same bee colony, and the consequences of such cell size variability.

## Figures and Tables

**Table 1:** Number of hemolymph samples in each age group of workers (1 d, 7 d, 14 d, and 21 d) in the three consecutive years.

Age	Group	Year		
		2020	2021	2022
1 day	SMC	15	15	15
	STC	15	15	15
7 days	SMC	15	15	15
	STC	15	15	15
14 days	SMC	15	15	15
	STC	15	15	15
21 days	SMC	-	15	15
	STC	-	15	15

SMC—workers reared in small-cell combs; STC—workers reared in standard-cell combs.

**Table 2:** Effect of the year (2020, 2021, and 2022) and age (1 d, 7 d, 14 d, and 21 d) on hemolymph parameters in workers reared in small- and standard-cell combs in laboratory cage tests.

Hemolymph Parameters	Impact of the Year		Impact of the Age					
			2020		2021		2022	
	SMC	STC	SMC	STC	SMC	STC	SMC	STC
protein concentration	H = 84.74	H = 89.99	F = 33.14	F = 31.64	H = 48.45	F = 48.66	H = 49.32	H = 48.87
	df = 2	df = 2	df = 2	df = 2	df = 3	df = 3	df = 3	df = 3
	*p* = 0.00	*p* = 0.00	*p* = 0.00	*p* = 0.58	*p* = 0.00	*p* = 0.00	*p* = 0.00	*p* = 0.00
activities of acidic proteases	H = 5.38	H = 4.82	H = 32.98	H = 34.26	H = 55.33	H = 55.32	H = 55.32	H = 50.95
	df = 2	df = 2	df = 2	df = 2	df = 3	df = 3	df = 3	df = 3
	*p* = 0.07	*p* = 0.09	*p* = 0.00	*p* = 0.00	*p* = 0.00	*p* = 0.00	*p* = 0.00	*p* = 0.00
activities of neutral proteases	H = 5.62	H = 0.90	H = 32.96	H = 33.19	H = 55.31	H = 55.23	H = 54.57	H = 55.33
	df = 2	df = 2	df = 2	df = 2	df = 3	df = 3	df = 3	df = 3
	*p* = 0.06	*p* = 0.63	*p* = 0.00	*p* = 0.00	*p* = 0.00	*p* = 0.00	*p* = 0.00	*p* = 0.00
activities of alkaline proteases	H = 4.82	H = 11.18	H = 37.15	H = 30.02	H = 55.32	H = 55.35	H = 54.55	H = 54.21
	df = 2	df = 2	df = 2	df = 2	df = 3	df = 3	df = 3	df = 3
	*p* = 0.09	*p* = 0.00	*p* = 0.00	*p* = 0.00	*p* = 0.00	*p* = 0.00	*p* = 0.00	*p* = 0.00
activities of acidic protease inhibitors	F = 28.14	H = 66.69	F = 51.14	F = 144.61	F = 507.88	H = 50.04	H = 55.33	F = 235.74
	df = 2	df = 2	df = 2	df = 2	df = 3	df = 3	df = 3	df = 3
	*p* = 0.00	*p* = 0.00	*p* = 0.00	*p* = 0.00	*p* = 0.00	*p* = 0.00	*p* = 0.00	*p* = 0.00
activities of neutral protease inhibitors	H = 90.98	F = 100.25	F = 54.11	F = 167.76	F = 679.91	H = 51.91	H = 55.23	H = 51.14
	df = 2	df = 2	df = 2	df = 2	df = 3	df = 3	df = 3	df = 3
	*p* = 0.00	*p* = 0.00	*p* = 0.00	*p* = 0.00	*p* = 0.00	*p* = 0.00	*p* = 0.00	*p* = 0.00
activities of alkaline protease inhibitors	H = 36.69	H = 71.08	H = 32.87	F = 139.04	F = 970.37	F = 219.27	F = 289.44	F = 142.65
	df = 2	df = 2	df = 2	df = 2	df = 3	df = 3	df = 3	df = 3
	*p* = 0.00	*p* = 0.00	*p* = 0.00	*p* = 0.00	*p* = 0.00	*p* = 0.00	*p* = 0.00	*p* = 0.00

SMC—workers reared on small-cell combs; STC—workers reared on standard-cell combs, H—value of statistics for the Kruskal–Wallis test; F—value of Fisher’s test for ANOVA; df—number of degrees of freedom; *p*—probability value. Impact of the year and impact of the age in SMC and STM groups are significant at *p* ≤ 0.01.

**Table 3:** Changes in the protein concentrations and activities of proteases and their inhibitors in workers reared in small-cell combs (SMC) in comparison with workers reared in standard-cell combs (STC) at the age of 1, 7, 14, and 21 days.

Hemolymph Parameters	2020				2021				2022			
	1 d	7 d	14 d	21 d	1 d	7 d	14 d	21 d	1 d	7 d	14 d	21 d
protein concentration	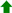	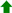	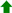	-	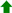	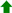	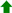	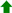	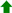	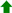	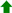	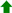
activities of acidic proteases	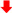	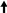 n.s.	= n.s.	-	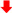	= n.s	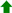	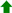	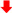	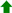	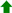	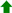
activities of neutral proteases	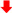	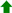	= n.s.	-	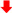	= n.s	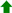	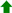	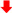	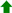	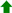	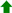
activities of alkaline proteases	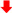	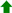	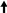 n.s.	-	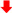	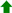	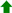	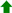	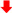	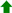	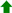	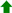
activities of acidic protease inhibitors	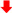	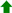	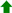	-	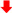	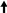 n.s.	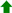	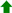	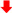	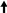 n.s.	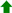	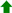
activities of neutral protease inhibitors	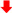	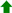	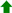	-	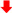	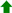	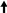 n.s.	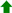	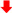	= n.s.	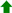	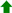
activities of alkaline protease inhibitors	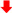	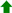	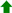	-	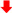	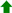	= n.s.	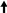 n.s.	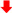	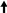 n.s.	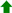	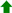

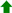
—green arrow indicates that the value of the trait is significantly higher in SMC workers than STC workers; 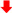
—red arrow indicates that the value of the trait is significantly lower in SMC workers than STC workers; n.s.—statistically insignificant difference between SMC workers and STC workers; 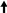
—black arrow indicates that the value of the trait is higher in SMC workers than STC workers but without statistical significance; =—equal sign indicates that the value of the trait in SMC workers is close or equal to the trait value in STC workers.

**Table 4:** Comparison of trends in the age-related changes to hemolymph parameters in workers reared in small-cell combs (SMC) and standard-cell combs (STC) as observed in the present laboratory tests and the apiary tests carried out by Dziechciarz et al. [30].

Hemolymph Parameters	SMC		STC	
	Laboratory	Apiary	Laboratory	Apiary
protein concentration	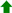	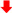	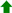	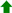
activities of acidic proteases	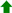	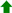	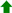	=
activities of neutral proteases	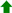	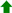	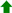	=
activities of alkaline proteases	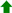	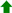	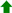	=
activities of acidic protease inhibitors	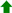	n.t.	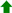	n.t.
activities of neutral protease inhibitors	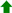	n.t.	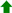	n.t.
activities of alkaline protease inhibitors	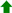	n.t.	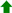	n.t.

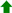
—green arrow indicates an upward trend in the value of the trait with age; 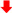
—red arrow indicates a downward trend in the value of the trait with age; =—equal sign indicates a value of the trait persisting at a similar level with age; n.t.—changes in the value of the trait do not showing similar trends with age.

## Data Availability

The data presented in this study are available on request from the corresponding author.
